# An urea, arginine and carnosine based cream (Ureadin Rx Db ISDIN) shows greater efficacy in the treatment of severe xerosis of the feet in Type 2 diabetic patients in comparison with glycerol-based emollient cream. A randomized, assessor-blinded, controlled trial

**DOI:** 10.1186/1471-5945-12-16

**Published:** 2012-09-25

**Authors:** Adalberto Federici, Giovanni Federici, Massimo Milani

**Affiliations:** 1 Servizio Podologia, Via Nomentana 27 Monterotondo, Rome, Italy; 2 Solo practice, Via A. Nota 18, 20126, Milan, Italy

**Keywords:** Skin xerosis, Diabetes, Urea, Controlled trial

## Abstract

**Background:**

Xerosis is a common skin disorder frequently observed in diabetic patients. An effective hydration of foot skin in diabetics is a relevant preventive strategy in order to maintain a healthy foot. Urea is considered an effective hydrating and emollient topical product. The aim of the present study was to evaluate the efficacy of topical urea 5% with arginine and carnosine *(Ureadin Rx Db, ISDIN Spain) (UC)* in comparison with glycerol-based emollient topical product *(Dexeryl, Pierre Fabre) (EC),* in Type 2 diabetic patients.

**Methods:**

We assessed the effect of UC on skin hydration in a randomized, evaluator-blinded comparative study in 40 type II diabetic patients, aged 40–75 years, treated with UC or the comparator for 28 days with a twice-daily application. The principal outcomes were the Dryness Area Severity Index (DASI) Score and the Visual Analogue Score (VAS) for skin dryness evaluated at baseline and at the end of study period by an investigator unaware of treatment allocation.

**Results:**

UC induced significantly greater hydration than EC with an 89% reduction in DASI score (from 1.6 to 0.2; p < 0.001) in comparison with baseline values. After 4 weeks, compared with the control group, DASI score in UC treated group was significantly lower (0.2 vs. 1.0; p = 0.048). VAS score (high values mean better hydration) significantly increased in both groups during treatment. VAS score at the end of treatment period was significantly higher in UC group in comparison with EC group (9.8 vs. 8.2; p = 0.05).

**Conclusion:**

Application of urea 5%, arginine and carnosine cream increases skin hydration and alleviates the condition of skin dryness in Type 2 diabetic patients in comparison with a control glycerol-based emollient product. (Dutch Trials Register trial number 3328).

## Background

Cutaneous complications are common in diabetes, with approximately 30% of patients experiencing some skin involvement during the course of their illness and these may also be an early indicator of undiagnosed diabetes [1]. In particular xerosis is a common skin disorder frequently observed in diabetic patients [2]. Skin xerosis and callous formation could be risk factors for diabetic ulcers developing [3]. An effective hydration of foot skin in diabetics is a relevant preventive strategy in order to maintain a healthy foot [4]. Emollient and moisturizer topical products are efficacious in repairing the epidermal barrier function and in ameliorating xerosis [5]. However few studies have been conducted in diabetic patients assessing wheter this treatment can help correct alterations in functional and mechanical properties of diabetic skin. Urea is considered an effective hydrating and emollient topical product [6]. Recent experimental data performed in human keratinocytes suggest that urea is not a simple emollient compound but it is able to improve cell differentiation increasing gene expression of transglutaminase, filaggrin, aquaporin, and loricrin, therefore improving keratinocytes differentiation [7]. Arginine is an important substrate for Nitric Oxide formation [8]. In diabetic skin a deficit in NO production has been demonstrated [9]. This reduction could be due to an enhanced arginine consumption linked to high arginase enzymatic activity [10]. Carnosine is able to interfere with advanced glycosylated end-products formation [11]. This action has been also demonstrated for urea [12]. Recently a topical cream product containing urea 5%, arginine and carnosine has been developed (Ureadin Rx Db, Isdin Spain). This formulation, from a theoretical point of view, is an interesting topical product with a composition particularly suitable for the specific treatment of the xerotic skin in diabetic patients. However, so far, not controlled clinical data are available particular with a head-to-head comparison design with standard topical emollient treatment. The aim of the present study was to evaluate the efficacy of topical urea *(Ureadin Rx Db, ISDIN)* containing also arginine and carnosine, in comparison with glycerol-based (15%) emollient topical product containing also vaseline (8%) *(Dexeryl, Pierre Fabre* ) in the treatment of xerotic skin in Type 2 diabetic patients.

## Methods

### Study design

The present study was a mono-centre prospective, parallel group, randomised, assessor-blinded trial. Randomisation list with a 1:1 ratio and with a block of 4 was generated by the mean of statistical software (G-Power). Study trial was registered in the Dutch Trials Register (*trial number 3328*). Local Institutional Review Board (Fitness Metabolic ONLUS Monterotondo) approved the study protocol. Study was performed between March 2011 and February 2012.

### Patient selection

Patients were enrolled in the trial after their written informed consent according to the Declaration of Helsinki [13]. Patients enrolled in this study were men and women, aged between 40 and 75 years, with a confirmed diagnosis of type 2 diabetes and moderate-to-severe foot xerosis, who had not used any topical moisturizers on their feet for at least 2 weeks. Insulin-dependent diabetes mellitus, presence of foot lesions and peripheral arterial diseases were the main exclusion criteria. We assessed the effect of urea 5% cream (UC) containing also arginine and carnosine on skin hydration in 40 type 2 diabetic patients treated with UC or the comparator (EC) for 28 days with a twice-daily application regimen. As comparator we have chosen a glycerol, vaseline and liquid paraffin cream (Dexeryl) which is commonly used as topical hydrating agent for the treatment of skin xerosis. Both treatments were applied on the feet (dorsal and plantar regions) and in the distal third of the leg.

### Study outcomes

The principal outcomes of the trial were the *Dryness Area Severity Index (DASI)* score, according to Serup et al. [14], and the *Visual Analogue Score (VAS) for skin dryness* evaluated at baseline and at the end of study period by an investigator unaware of treatment allocation (GF). The DASI score evaluates dry skin using a 5-point Likert scale ranging from 0 (=no dryness) to 4 (=severe dryness). We used a visual analogue scales (VAS) for subjective evaluation of dryness of the skin; the results were converted to a points scale on which 0 denoted extreme skin dryness and 10 the best skin hydration state imaginable. Secondary endpoints of the study were the percentage reduction of DASI score in comparison with baseline values and the evolution of patient-assessed itch sensation according to Hagermark [15] with a scale from 0 (extreme itch sensation) to 10 (not itch).

### Statistical analysis

Statistical analyses were performed using SPSS statistical software (ver. 13.0). Data were expressed as mean (SD). All P values were two-sided. The present trial was designed as a superiority trial. The power calculation assumed a difference between the two treatments in the DASI score at week 4 of at least 1.1 points with an effect size of 0.6. This assumption provided 90% power at an alpha level of .05 (two-tailed test) for a sample size of at least 40 evaluable patients in total. Sample size calculation was performed using G*Power program Ver.3.03 (Kiel, Germany). Two-tailed unpaired T-test, two-tailed Mann–Whitney (unpaired) and Wilcoxon (paired) tests were applied to compare treatments and to compare baseline levels with values at the end of study period. The analysis was based on the *intention-to- treat principle* and involved all patients who were randomly assigned to the treatments. A P value ≤0.05 was considered statistically significant.

## Results

**Figure 1:**
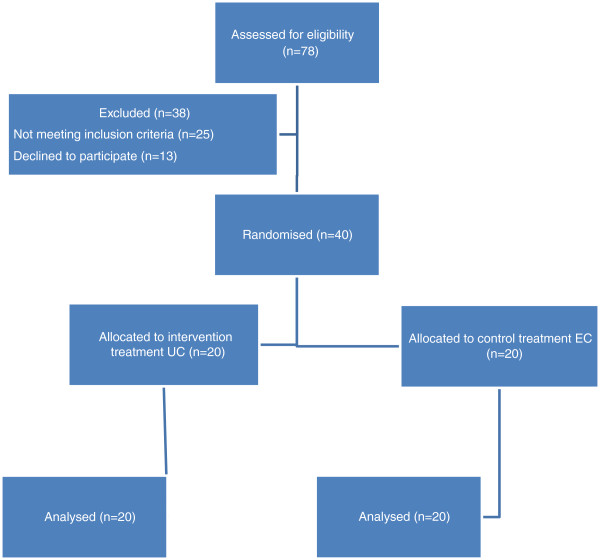
Study flow diagram.

**Figure 2:**
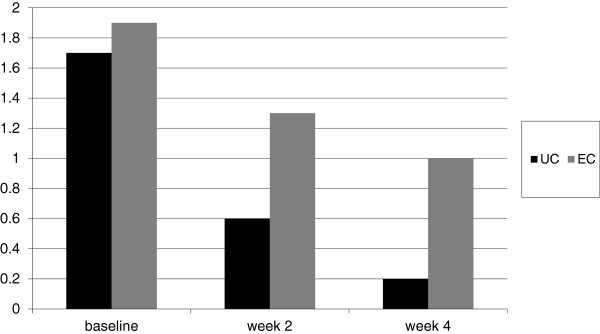
**Evolution of DASI score (mean values) from baseline at week 2 and week 4 in UC treated patients and EC treated group.** Lower scores mean reduction in xerosis.

**Figure 3:**
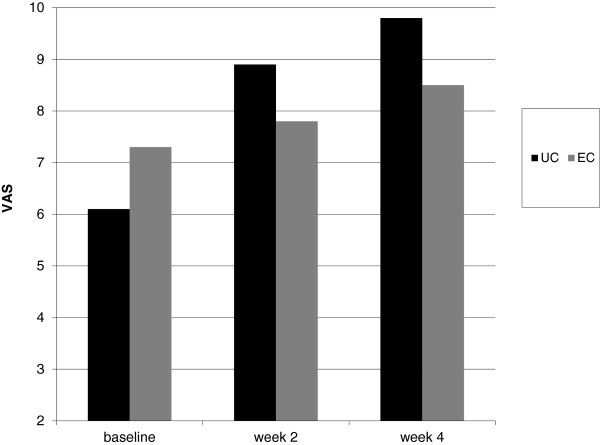
**Evolution of VAS score (means values) for skin xerosis from baseline at week 2 and week 4 in UC treated patients and EC treated group.** High score means less xerosis.

**Figure 4:**
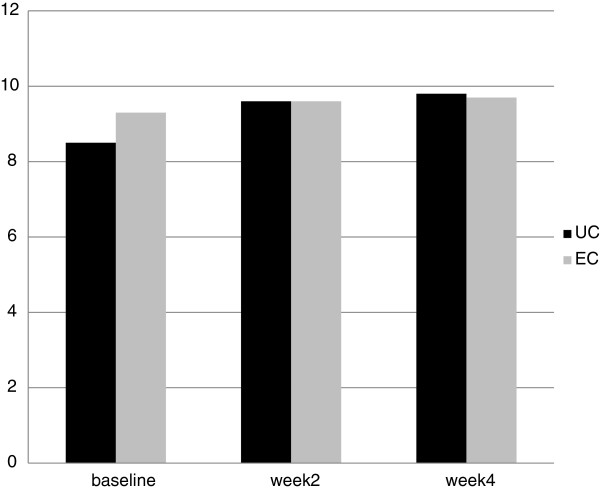
**Evolution of Itch score (IS) (means values) from baseline at week 2 and week 4 in UC treated patients and EC treated group.** High score means less itch.

A total of 78 patients were screened for inclusion in the study. A total of 40 patients, fulfilling inclusion and exclusion criteria were enrolled: 20 were randomised to UC and 20 to EC group. Table 1 shows the patients characteristics at baseline. Main characteristics at randomisation were similar in the two groups even if there werea significative differences in patients randomised to UC in comparison to EC group regarding age, duration of diabetes and baseline VAS scores mean values. All patients concluded the 4-week treatment period. Figure 1 shows the study flowchart. UC induced a significantly greater hydration than the glycerol-based emollient cream: in UC group DASI score was reduced from 1.7 at baseline to 0.2 at week 4 ( p < 0.001; Wilcoxon test, a percentage reduction of 89%); in the EC group DASI score was reduced from 1.9 to 1.0 at week 4, a 47% percentage reduction in comparison with baseline values. After 4 weeks, compared with the control group, mean DASI score in UC treated group was significantly lower (0.2 vs. 1.0; p = 0.048, unpaired T-test) (Figure 2). Median values of DASI score were 1.5 in UC group and 2 in EC at baseline. At week 4 median DASI scores were 0 for UC group and 1 in EC group, respectively (p = 0.0005 Mann–Whitney test). Mean VAS score significantly improved in both groups during treatment. Mean VAS score at the end of treatment period was significantly higher in UC group in comparison with the control group (9.8 vs. 8.2; p = 0.05, unpaired T-Test) (Figure 3). Median values of VAS score were 6 in UC group and 7 in EC at baseline. At week 4 median VAS scores were 10 for UC group and 9 in EC group, respectively(p = 0.0001 Mann–Whitney test). Mean Itching score (IS) at baseline were 8.5 in UC and 9.3 in EC group. At week 4, mean IS increased significantly (P = 0.05, Wilcoxon test) in UC to 9.9 in comparison to baseline. In EC group mean IS score was 9.7 at week 4. No differences were observed in the two groups at week 4 (Figure 4).

**Table 1:** Patients characteristics at randomization, data presented as mean (SD)

**Variable**	**UC group (n = 20)**	**EC group (n = 20)**	**P values**
Men/women	10/10	6/14	0.7
Age, years	66 (7)	58 (8)	0.004
History of Diabetes, years	14 (6)	9 (3)	0.03
Serum glucose mg/100 mL	153 (40)	153 (21)	0.6
DASI	1.7 (0.8)	1.9 (0.5)	0.5
VAS skin xerosis	6.1 (1.4)	7.3 (1.2)	0.007
Itching score (from 0 to 10)	8.5 (0.6)	9.3 (0.9)	0.5

## Discussion

Diabetes mellitus induces many pathophysiologic changes in the skin [16]. Diabetes also induces increase in the dermis of advanced glycosylation end products (AGEs), which may be responsible for some skin changes in persons with elevated blood sugars [17]. Xerosis (with prevalence higher than 40%) with pruritus and scleroderma-like skin changes are the most commonly observed cutaneous manifestations of this common disease [18]. Xerosis of the diabetic foot could promote ulceration through the development of fissures and hyperkeratosis. Its treatment is therefore important and must be implemented early on. Skin xerosis is commonly treated with the repeated use of emollients and moisturizers [19]. Their use is based on sound evidence of the importance of maintaining the skin's water content. Emollient products could vary in term of beneficial effects on the skin [20]. Recent data have shown that topical urea can act not only as a simple hydrating molecule but in addition it could be able to improve cell differentiation of keratinocytes. In a randomized placebo controlled trial Garrigue [21] et al. have shown that topical urea 5% is able to improve xerosis in the diabetic foot by 61% after 4 weeks of treatment. In our study we compared urea, arginine and carnosine topical product with a standard treatment reference (Dexeryl). Reduction of xerosis score observed in our trial was 90% in the UC group. This clinical effect could be due to the particular composition of UC. Urea and carnosine could favourably interfere with formation and accumulation of advanced glycated end-products [22]. Topical urea is able to increase the synthesis of aquaporin in keratinocytes therefore increasing the hydration of the skin [6]. Arginine supplementation improves microcirculation in diabetes [23]. Therefore UC composition could have a strong rational as an “active” emollient for the treatment of skin xerosis observed in diabetic patients.

## Conclusion

Our study has shown that application of urea 5% associated with arginine and carnosine cream increase skin hydration and alleviate the condition of skin dryness in Type 2 diabetic patients in comparison with a control glycerol-based emollient product. Some limitations should be considered in evaluating our results. First this study was not double-blind. The main difficulty in performing a double-blind trial in this setting was linked to the different formulations and texture of the study products. Therefore we decided to perform an assessor-blinded evaluation of the primary endpoint of the study. A second limitation of our study is that we have evaluated as primary endpoint a subjective clinical assessment parameters (DASI score and the VAS) instead of an instrumental objective variable. However the main therapeutic goal of emollient treatment in diabetes is to obtain an increase in hydration of the skin clinically evaluable. Further studies are necessary to evaluate if the treatment with this topical product could be associated with improvement in microcirculation and/or modification of skin structure in diabetes.

## Competing interests

The authors declare that they have no competing interests.

## Authors’ contributions

AF and GF had the original study idea and participated in its design and coordination. MM helped regarding study design, protocol definition, data collection and analysis. AF and GF carried out the patients selection and visits. All authors read and approved the final manuscript.

## Pre-publication history

The pre-publication history for this paper can be accessed here:

http://www.biomedcentral.com/1471-5945/12/16/prepub
